# Association Between *Plasmodium* Infection and Blood Count Values: Implications for Malaria Diagnosis in Resource-Limited Settings

**DOI:** 10.1007/s11686-024-00972-2

**Published:** 2025-01-28

**Authors:** Laura Seijas-Pereda, Pablo Fernández-González, Isaac Asare, Godlove Osei Asumang, Emmanuel Frimpong, Carlos Rescalvo-Casas, Marcos Hernando-Gozalo, Ramón Pérez-Tanoira

**Affiliations:** 1 Department of Biomedicine and Biotechnology, Faculty of Medicine, University of Alcala, Alcala de Henares, Spain; 2 Department of Microbiology, Principe de Asturias University Hospital, Alcala de Henares, Spain; 3 Department of Dermatology, Ramón y Cajal University Hospital, Madrid, Spain; 4 Haematology Laboratory, Saint Dominic Hospital, Akwatia, Eastern-Region, Ghana; 5 Department of Organic and Inorganic Chemistry, University of Alcala, Alcala de Henares, Spain

**Keywords:** Ghana, Parasitaemia, Haematology, Laboratory test, Tropical disease

## Abstract

**Purpose:**

Malaria remains a major global health challenge, particularly in sub-Saharan Africa and low- and middle-income countries (LMICs), contributing substantially to mortality and morbidity rates. In resource-limited settings, access to specialized diagnostic tests is often restricted, making basic blood analysis a valuable diagnostic tool. This study investigated the correlation between malaria infection and full blood count values in a rural region of Ghana during the 2022 rainy season, aiming to highlight diagnostic insights available from routine blood analyses.

**Methods:**

A retrospective case-control analysis was conducted on 544 confirmed malaria cases, comparing their blood values with those of matched malaria-negative controls.

**Results:**

Parasitaemia levels peaked during the rainy season, with July showing the highest values. Malaria-positive patients exhibited lower levels of haemoglobin, white blood cells, lymphocytes, and platelets, but higher neutrophil counts compared to controls. Middle-aged women had significantly lower haemoglobin levels than men, and younger individuals showed higher parasitaemia levels. A negative correlation was found between *Plasmodium* density and haemoglobin and platelet counts, while positive correlations were observed with white blood cell and neutrophil counts.

**Conclusion:**

This study highlights the significant burden of malaria in rural Ghana during the rainy season and underscores the impact of infection on blood values. Routine full blood count analysis provides a practical and accessible diagnostic tool in resource-limited settings. The findings emphasise the importance of targeted interventions for high-risk groups, particularly children and women, to improve patient management and reduce malaria-related morbidity.

**Graphical Abstract:**

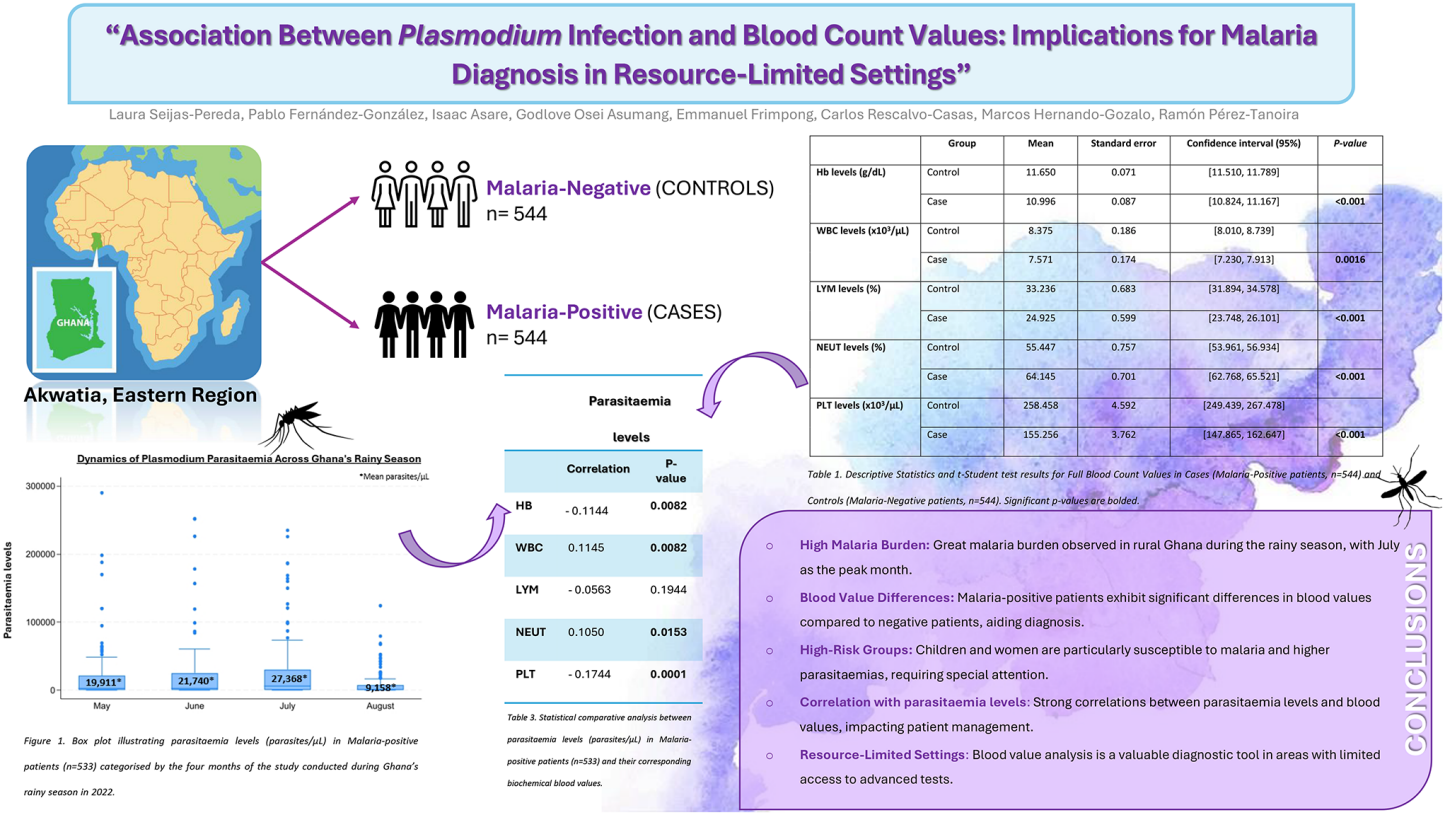

## Introduction

Malaria is a parasitic disease caused by the protozoan *Plasmodium* sp., transmitted through the bite of female Anopheles mosquitoes. Among the parasite species affecting humans, *Plasmodium falciparum* (*P. falciparum*) poses the greatest threat. It is strongly associated with severe malaria across all age groups and accounts for the highest morbidity and mortality rates related to the disease, particularly in sub-Saharan Africa [1, 2]. Globally, there were an estimated 247 million reported cases of malaria in 2021 across 84 endemic countries. The African Region accounted for approximately 95% of these cases, leading to an alarming 593,000 estimated malaria-related deaths in 2021. This disease also continues to be a significant cause of mortality among children under 5 years of age in Africa, claiming the lives of approximately 1,200 African children daily [2–4].

Our study is conducted in this pivotal setting, specifically in Ghana, an endemic country for malaria where perennial transmission affects individuals of all age groups [5]. In endemic areas, malaria often emerges as the primary cause of fever, but its symptoms, such as fever, headache, shaking chills, muscle pains, and digestive manifestations can be nonspecific and mistaken for other illnesses [1, 6, 7]. Severe falciparum malaria is typically fatal and age-dependent, frequently presenting with severe anaemia and various manifestations of multi-organ damage [6, 8].

In numerous regions of Africa, the onset of the seasonal rains heralds a rapid increase in hospital admissions within weeks. During this period, a significant number of patients will be grappling with *P. falciparum* malaria, coinciding with the life cycles of mosquitoes [3, 9]. The host immune response to malaria infection is extensively studied but not fully understood. Biochemical blood values have been observed to interact with the disease; for instance, anaemia, prevalent in this population, is believed to confer protection against severe falciparum malaria [10–13], and high parasitaemia has been seen to be inversely correlated with platelet count [14, 15]. However, further studies are warranted to establish specific correlations between blood values and malaria diagnosis.

In settings like Ghana, classified as low- and middle-income countries (LMICs), access to specific laboratory diagnostic tests is often limited. However, basic blood component analysis is generally available, and recognizing alterations in these parameters during disease can provide valuable diagnostic insights and aid in risk prediction [16, 17]. Therefore, our objective is to study malaria infection in a rural region of Ghana while exploring the correlation between full blood count values and the disease. To achieve this, we will compare malaria-positive and negative patients from the same setting, aiming to identify significant differences in their blood values that can guide patient diagnosis in locations where specialized tests may be unavailable.

## Materials and Methods

### Study, Location and Period

This retrospective case-control study was conducted at the haematology laboratory of Saint Dominic Hospital in Akwatia, located in the Eastern Region of Ghana, a rural zone situated 124 km from Accra, the country’s capital. Saint Dominic Hospital serves as the primary medical referral centre for the region, equipped with 320 beds and attending to approximately 80,000 residents. The hospital is complemented by several smaller healthcare centres in its vicinity. The laboratory within the hospital is well-equipped, housing all necessary basic materials, machinery, and a proficient team of trained professionals, including physicians and technicians.

Our study was conducted during Ghana’s rainy season, spanning four months in 2022 (May, June, July, and August). This timeframe was chosen to coincide with the peak of malaria infections in the region.

### Population

The study enrolled individuals with suspected malaria, referred by clinicians for testing at the hospital’s haematology laboratory. All patients testing positive for malaria during the specified timeframe and undergoing blood value analysis were included. Negative controls were carefully matched to each positive patient based on diagnosis date, sex, and age, and underwent the same blood analyses. Thus, two distinct groups were established: cases, consisting of patients with confirmed malaria, and controls, comprising individuals with negative results. No personal or traceable data were collected; only information such as sex, age, month of diagnosis, biochemical blood values, and *Plasmodium* parasitaemia levels were recorded.

### Specimens Processing

For malaria testing, a drop of patient’s blood was used for the preparation of both thick and thin blood films, which were created following the guidelines established by the World Health Organization and appropriately labelled. The thin films were fixed with absolute methanol, and both thick and thin smears were stained using a 10% Giemsa stain. After thorough drying of the slides, qualified staff diagnosed malaria infection by detecting, identifying, and quantifying malaria parasites in blood films using light microscopy.

For the biochemical blood test, a full blood count analysis was performed using the Sysmex Analyser XN-350™. Measurements were collected for Haemoglobin (Hb; g/dL), White Blood Cells (WBC; x10^3^/µL), Lymphocytes (LYM; %), Neutrophils (NEUT; %) and Platelets (PLT; x10^3^/µL). Thrombocytopenia was defined as platelets count of less than 150 × 10^3^/µL [18].

### Statistical Analysis

Statistical analysis was conducted using STATA/MP 18.0 (StataCorp, Texas, USA). Continuous variables were presented as median and interquartile ranges (IQR), while categorical variables were presented as proportions unless otherwise specified. Group differences were assessed using appropriate statistical tests, including the Mann-Whitney U-test, t-Student test, χ2 test, or Fisher’s exact test. All p-values were calculated in a two-tailed manner, and significance was defined as *p*-value ≤ 0.05. To assess the correlation between different variables, various correlation tests were employed, including Pearson correlation and scatter diagrams.

## Results

### Population

During the four-month study period, 1088 paired patients were enrolled, with 544 classified as cases (malaria-positive) and 544 as controls (malaria-negative). Each comparative group comprised 204 men (37.5%) and 340 women (62.5%). Patient’s ages ranged from 0.8 years to 96, with 19 as the median (IQR 8–34).

The entire study population was stratified into five age groups compromising: 194 children under five (17.8%), 212 paediatrics between 5 and 14 (19.5%), 252 young individuals between 15 and 24 (23.2%), 260 adults aged 25–44 years (23.9%), and 170 individuals aged 45 years and above*.

The distribution of cases during this timeframe was: 137 positive patients in May (25.18%), 133 in June (24.45%), 144 in July (26.47%) and 130 in August (23.90%). Controls were accordingly selected.

*(The number of males and females from each age group can be seen in Table 2).

### Biochemical Blood Values

Following the acquisition of full blood count values from the patients, a comparative analysis was undertaken between the two groups stratified by malaria infection status (cases and controls). Table 1 provides descriptive statistics and the results of the t-Student test, demonstrating significant differences between the malaria-positive and negative patients across the studied biochemical blood values.

**Table 1:** Descriptive statistics and t-Student test results for full blood count values in cases (malaria-Positive patients, *n* = 544) and controls (Malaria-Negative patients, *n* = 544). Haemoglobin (hb), White blood cells (WBC), lymphocytes (LYM), Neutrophiles (NEUT) and platelets (PLT). Significant p-values are bolded

	Group	Mean	Standard error	Confidence interval (95%)	*P*-value
Hb levels (g/dL)	Control	11.650	0.071	[11.510, 11.789]	
	Case	10.996	0.087	[10.824, 11.167]	**< 0.001**
WBC levels (x10^3^/µL)	Control	8.375	0.186	[8.010, 8.739]	
	Case	7.571	0.174	[7.230, 7.913]	**0.0016**
LYM levels (%)	Control	33.236	0.683	[31.894, 34.578]	
	Case	24.925	0.599	[23.748, 26.101]	**< 0.001**
NEUT levels (%)	Control	55.447	0.757	[53.961, 56.934]	
	Case	64.145	0.701	[62.768, 65.521]	**< 0.001**
PLT levels (x10^3^/µL)	Control	258.458	4.592	[249.439, 267.478]	
	Case	155.256	3.762	[147.865, 162.647]	**< 0.001**

The statistical analysis reveals significantly lower levels in most of the studied blood values in malaria-positive patients, including Haemoglobin, White Blood Cells, Lymphocytes, and Platelets. Notably, Neutrophils are the only parameter tested that exhibits significantly higher levels in Malaria-positive patients.

Thrombocytopenia was defined as a platelet count < 150,000/µL [18, 19]. Mean platelet levels in the Malaria-positive group closely approached this definition, with 297 of these patients exhibiting thrombocytopenia (54.6%). In contrast, in the control group, only 97 patients (17.8%) manifested this condition.

### Malaria Positive Patients

After establishing the significant differences between the two comparative groups, we focused on the blood values of the malaria-positive population. Throughout the study, we accumulated a total of 544 positive malaria cases during the established period of Ghana’s 2022 rainy season. All diagnoses were confirmed indicating *P. falciparum* infection. Table 2 presents the biochemical characteristics and *Plasmodium* levels of the malaria-positive population separated by sex and age group. Notably, it illustrates a decline in parasite density while advancing age, with high parasitaemia densities observed until adulthood. Interestingly, beyond paediatric ages, this density is higher in women compared to men, exhibiting an inverse correlation with haemoglobin levels.

**Table 2:** Mean biochemical parameters and plasmodium parasitaemia levels of the positive-malaria population (*n* = 544) classified by sex and age group. Blood values studied include Haemoglobin (Hb), White blood cells (WBC), lymphocytes (LYM), Neutrophiles (NEUT) and platelets (PLT)

Age groups	Sex	Hb levels (g/dL)	WBC levels (x10^3^/µL)	LYM levels (%)	NEUT levels (%)	PLT levels (x10^3^/µL)	Parasitaemia density	TOTAL (*n*)
< 5	Male	9.53	10.12	39.07	56.56	161.90	58392.52	51
	Female	9.42	10.54	38.30	51.72	147.70	33424.70	46
5—14	Male	10.70	20.91	25.22	68.05	150.95	29411.05	65
	Female	10.57	8.49	23.40	66.90	159.39	18325.95	41
15—24	Male	13.06	6.90	19.16	69.36	137.27	14896.00	41
	Female	10.73	6.98	19.64	68.98	142.88	18157.18	85
25—44	Male	13.16	7.16	26.98	58.46	174.48	6138.15	26
	Female	10.93	6.70	22.98	66.42	165.24	6295.34	104
> 45	Male	12.41	6.20	25.25	62.04	173.38	4528.50	21
	Female	11.71	6.34	25.75	62.91	155.09	8794.89	64

**Fig. 1:**
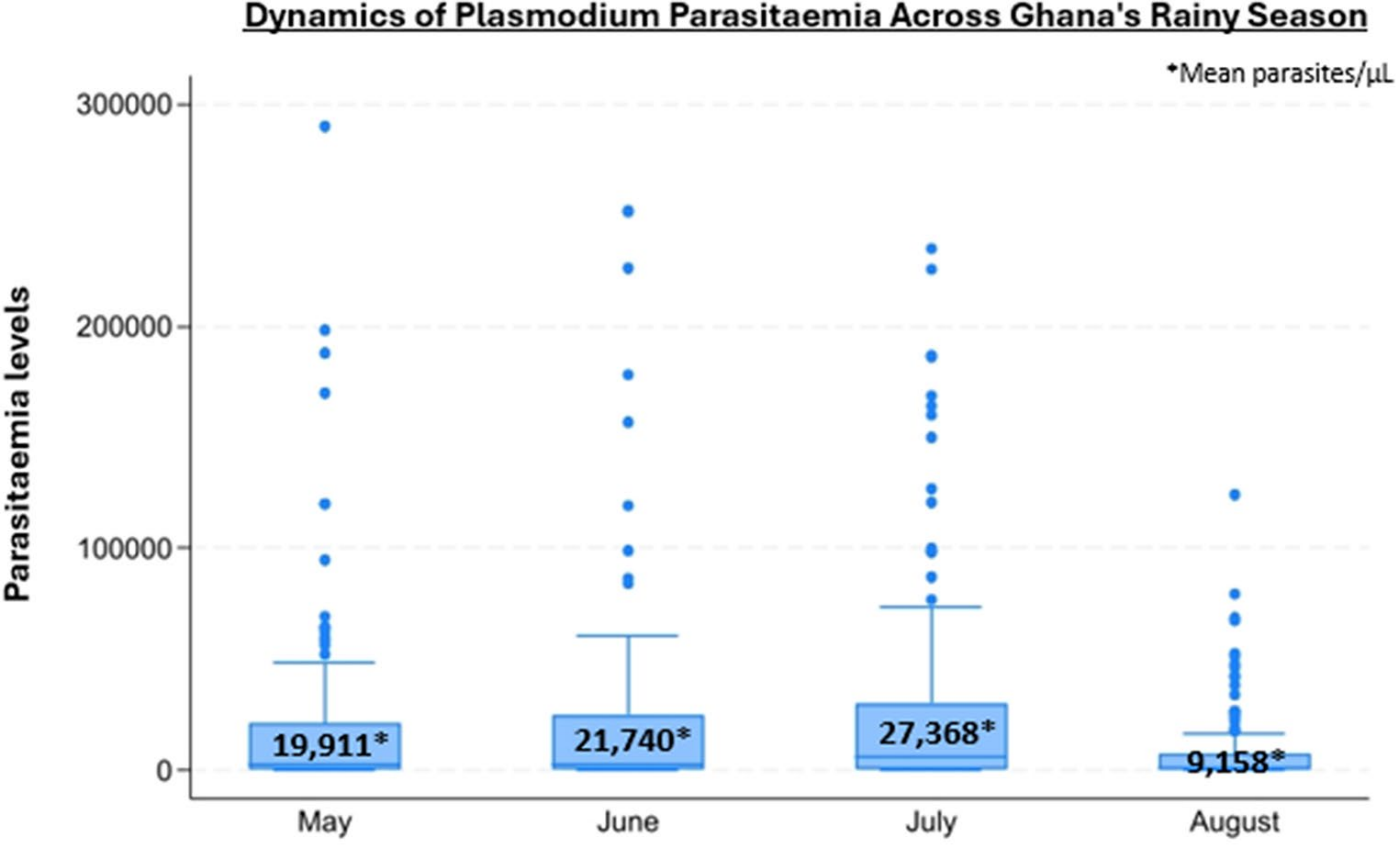
Box plot illustrating parasitaemia levels (parasites/µL) in Malaria-positive patients (*n* = 533) categorised by the four months of the study conducted during Ghana’s rainy season in 2022

Parasitaemia levels were assessed in 533 malaria-positive patients (98.0%), with a mean of 19,753.77 parasites/µL (± 40,288.98 par/µL). Figure 1 illustrates the parasitaemia levels categorised by the months of Ghana’s rainy season in 2022, showing that in our study setting and period, malaria parasitaemia densities were averaged and exhibited a mostly uniform distribution. Remarkably, only 23 patients had more than 100,000 parasites/µL. July exhibited the highest average parasitaemia levels, reaching 27,368.10 parasites/µL, while August was the sole month where the mean parasitaemia was lower than 10,000 parasites/µL.

Finally, we explored the associations between the patient’s *Plasmodium* levels and the studied biochemical blood values, as presented in Table 3.

**Table 3:** Statistical comparative analysis between parasitaemia levels (parasites/µL) in Malaria-positive patients (*n* = 533) and their corresponding biochemical blood values: Haemoglobin (Hb), White blood cells (WBC), lymphocytes (LYM), Neutrophiles (NEUT) and platelets (PLT). Level of correlation and its associate p-value are represented

		HB	WBC	LYM	NEUT	PLT
Parasitaemia levels	Correlation	-0.1144	0.1145	-0.0563	0.1050	-0.1744
	P-value	**0.0082**	**0.0082**	0.1944	**0.0153**	**0.0001**

The statistical analysis reveals significant correlations between parasitaemia levels and four of the analysed biochemical blood values. A negative correlation is observed between *Plasmodium* density and the levels of Haemoglobin and Platelets, indicating that an escalation in parasitaemia corresponds to a reduction in these parameters. Conversely, a positive correlation is noted with White Blood Cells and Neutrophils, suggesting that an increase in *Plasmodium* levels is associated with elevated counts of these parameters.

## Discussion

In this study, our objective was to explore the correlation between the presence of *Plasmodium* and full blood count values across diverse demographics in a rural region of Ghana. Situated within the WHO African region, Ghana is identified by the World Malaria Report 2022 as one of the countries most heavily burdened by malaria [2]. This was evidenced by our identification of 544 confirmed malaria cases, characterised by a mean high parasitaemia, in a specific geographic region of Ghana during a relatively short period. Here, we observed a gradual but discernible increase in the number of malaria-positive patients and parasitaemia levels as the rainy season progressed towards its peak, particularly in July, where the mean parasitaemia reached 27368.10 parasites/µL (Fig. 1). This trend reflects the dynamic nature of malaria transmission, with infection rates and parasitaemia levels fluctuating over time. It holds practical implications for patient management, as the likelihood of positive and severe cases is influenced by the month. Notably, malaria tends to exhibit higher prevalence in resource-limited settings like ours, rural areas within LMICs, where access to healthcare and diagnostic resources is often constrained [2, 13, 20].

Our cohort of patients also underscores the healthcare impact and frequency of malaria in our setting, highlighting the critical importance of utilising resources and elements, such as blood values analysis, that can help guide accurate diagnosis [9, 14]. In that direction, we found significant differences between the positive and negative malaria patients, with notably lower levels in most of the studied blood values among the malaria-positive population, including Haemoglobin, White Blood Cells, Lymphocytes, and Platelets. This aligns with findings from several studies indicating that low haemoglobin levels, and even anaemia, are very prevalent in positive patients and are also associated with severe malaria [6, 13, 21]. Coagulation abnormalities, particularly low platelets count, are also common in malaria infections, and the literature supports their role as a reliable indicator [14, 19, 22]. White blood cells are a central component of the immune system, and their distributions and quantities reflect the body’s response to infection [17, 23]. Regarding Lymphocyte count, we observed a significant depletion in malaria patients, consistent with findings from other studies that also report an association with mortality [17, 24, 25]. On the other hand, Neutrophils were the only studied parameter exhibiting significantly higher levels in the malaria-positive population, a trend also noted in the literature [23–25]. This isolated elevation is likely attributable to their function in pathogen clearance upon activation [26]. In summary, our findings support the existence of significant disparities in the studied blood values between malaria-positive and negative patients. It is noteworthy that abnormalities in blood values can often result from recurrent malaria infections, emphasising the importance of understanding the context and population when interpreting such results [6, 14, 22]. Should blood values appear normal, especially those specified, clinicians ought to reconsider their diagnosis and explore alternative causes and treatments. Proper management of malaria infection not only saves lives but also mitigates the development of parasitic resistance to malaria treatment and reduces the burden of disease [4, 27].

On the other hand, our analysis revealed a higher proportion of women within the malaria-positive population compared to men (62.5% vs. 37.5%), potentially attributed to women’s heightened propensity to seek healthcare assistance. However, epidemiological studies indicate that men exhibit a higher susceptibility to malaria infections due to their daily habits, despite often experiencing less severe or asymptomatic cases [1, 28]. Notably, middle-aged women exhibited significantly lower haemoglobin levels compared to men, which has been reported as protective against malaria infection but poses a significant risk during pregnancy [2, 6, 10]. Additionally, parasitaemia levels were notably higher among younger individuals, likely due to the ongoing development of their immune systems. It is well-documented that children in malaria-endemic regions of Africa often experience multiple episodes of uncomplicated malaria before acquiring protective immunity; however, some may progress to severe malaria [1, 9]. Subsequent examination of the association between parasitaemia levels and blood values indicated that higher parasitaemia correlated with reduced haemoglobin levels and platelet counts. Given the pre-existing lower levels of these parameters in women and the elevated parasitaemia levels observed in children, special attention should be directed towards these high-risk population groups, as disease progression to severe forms could rapidly occur. Monitoring blood values is imperative for malaria diagnosis, particularly in vulnerable populations such as the ones stated, where timely and appropriate management can significantly impact outcomes [2, 4].

### Limitations

The limitations of this study are multifaceted. Firstly, its retrospective design constrained the availability of detailed clinical data and hindered the capacity for conducting further analysis. Moreover, the reliance on clinician requests and patient resources for malaria analysis, owing to healthcare being fee-based, introduces a potential source of bias. Additionally, the exclusive use of microscopy as the diagnostic tool for malaria may have limited diagnostic accuracy, as it is subject to variations in performance influenced by personnel experience.

Furthermore, the study’s data stem from a hospital setting in a rural area, thereby potentially limiting its generalizability to other geographical regions with different epidemiological and socioeconomic contexts. Additionally, the inherent selection bias associated with data derived solely from patients seeking medical care at the hospital must be acknowledged.

## Conclusions

Our findings underscore the significant burden of malaria in a rural area of Ghana, particularly during the rainy season, with July emerging as the peak month for positive cases and parasitaemia levels. Noteworthy differences in blood values were observed between malaria-positive and negative patients. Furthermore, our study revealed variations in the impact of malaria by gender and age, emphasising the need for targeted attention to high-risk populations, especially children and women, who are more susceptible to malaria and its severe complications. Additionally, significant correlations were found between parasitaemia levels and blood values.

These findings highlight the diagnostic relevance of blood component states during malaria and demonstrate how basic laboratory tests, such as blood value analysis, can aid in accurate diagnosis. It is crucial to consider other potential causes and treatments in suspected malaria cases with normal blood values. These insights have practical implications for malaria management and patient diagnosis, particularly in resource-limited settings where advanced testing may not be available. Healthcare providers must remain vigilant, especially as the rainy season peaks, to ensure timely intervention and management of malaria infections, thereby reducing the risk of severe cases and alleviating the overall disease burden.

## Data Availability

No datasets were generated or analysed during the current study.
